# Catalase (CAT) Gene Family in Rapeseed (*Brassica napus* L.): Genome-Wide Analysis, Identification, and Expression Pattern in Response to Multiple Hormones and Abiotic Stress Conditions

**DOI:** 10.3390/ijms22084281

**Published:** 2021-04-20

**Authors:** Ali Raza, Wei Su, Ang Gao, Sundas Saher Mehmood, Muhammad Azhar Hussain, Wenlong Nie, Yan Lv, Xiling Zou, Xuekun Zhang

**Affiliations:** 1 Key Lab of Biology and Genetic Improvement of Oil Crops, Oil Crops Research Institute, Chinese Academy of Agricultural Sciences (CAAS), Wuhan 430062, China; alirazamughal143@gmail.com (A.R.); 82101181063@caas.cn (W.S.); gaoang1696555087@163.com (A.G.); snookas.saher90@gmail.com (S.S.M.); azharhussain301@gmail.com (M.A.H.); 2 Crops Research Laboratory, Xiaogan Academy of Agricultural Sciences, Xiaogan 432000, China; 15871339003@163.com; 3 College of Agriculture, Engineering Research Center of Ecology and Agricultural Use of Wetland of Ministry of Education, Yangtze University, Jingzhou 434025, China; zhang.xk@139.com

**Keywords:** abiotic stress, catalase, developmental tissues, gene structure, gene ontology, miRNA, plant hormones, ROS, stress responses

## Abstract

Catalase (CAT) is an antioxidant enzyme expressed by the *CAT* gene family and exists in almost all aerobic organisms. Environmental stresses induce the generation of reactive oxygen species (ROS) that eventually hinder plant growth and development. The CAT enzyme translates the hydrogen peroxide (H_2_O_2_) to water (H_2_O) and reduce the ROS levels to shelter the cells’ death. So far, the *CAT* gene family has not been reported in rapeseed (*Brassica napus* L.). Therefore, a genome-wide comprehensive analysis was conducted to classify the *CAT* genes in the rapeseed genome. The current study identified 14 *BnCAT* genes in the rapeseed genome. Based on phylogenetic and synteny analysis, the *BnCATs* belong to four groups (Groups I–IV). A gene structure and conserved motif analysis showed that Group I, Group II, and Group IV possess almost the same intron/exon pattern, and an equal number of motifs, while Group III contains diverse structures and contain 15 motifs. By analyzing the *cis*-elements in the promoters, we identified five hormone-correlated responsive elements and four stress-related responsive elements. Further, six putative bna-miRNAs were also identified, targeting three genes (*BnCAT4, BnCAT6,* and *BnCAT8*). Gene ontology (GO) enrichment analysis showed that the *BnCAT* genes were largely related to cellular organelles, ROS response, stimulus response, stress response, and antioxidant enzymes. Almost 10 *BnCAT* genes showed higher expression levels in different tissues, i.e., root, leaf, stem, and silique. The expression analysis showed that *BnCAT1–BnCAT3* and *BnCAT11–BnCAT13* were significantly upregulated by cold, salinity, abscisic acid (ABA), and gibberellic acid (GA) treatment, but not by drought and methyl jasmonate (MeJA). Notably, most of the genes were upregulated by waterlogging stress, except *BnCAT6, BnCAT9*, and *BnCAT10*. Our results opened new windows for future investigations and provided insights into the *CAT* family genes in rapeseed.

## 1. Introduction

Numerous environmental factors, including abiotic and biotic stresses, significantly limit plants’ growth and development [1]. Under stress environments, the plant modifies its homeostatic apparatus by producing a surplus level of reactive oxygen species (ROS). ROS, which consist of a superoxide anion, hydrogen peroxide, hydroxyl radical, and singlet oxygen, are produced as a lethal derivative of regular oxygen metabolism and serve as a signaling component in plant molecular biology [2,3]. Over the past decade, many investigations have discovered that ROS homeostasis is important for upholding normal cellular roles [2,3]. Therefore, upholding a considerable amount of ROS is crucial for appropriate cellular ROS signaling, and this amount can be attained by maintaining the balance among ROS generation and scavenging [2,3].

The major ROS-metabolizing enzymes include superoxide dismutase, catalase, peroxidase, glutathione peroxidase, ascorbate peroxidase, glutathione reductase, glutathione S-transferase, etc., among which the catalase (CAT; EC 1.11.1.6) enzyme is crucial for ROS depollution throughout stress, and it does not need cellular reductants as they mainly catalyze a dismutase retort [4]. The tetrameric haem-comprising CAT enzyme quickly decays H_2_O_2_, producing water (H_2_O) and oxygen (O_2_), and exists in almost all aerobic organisms [4]. The activity of CAT has been detected in peroxisomes, mitochondria, cytosol, and chloroplast [2,4]. Notably, CAT owns the extreme turnover proportion, and 26 million H_2_O_2_ molecules can be changed by one CAT molecule within one minute [5,6]. In animals, CAT enzymes are programmed by an exclusive *CAT* gene. In contrast, in plants, these enzymes are programmed by a gene family and have been systematically investigated in a number of plant species. For instance, one *CAT* family member (*CAT1*) has been reported in castor bean (*Ricinus communis* L.) [7], tomato (*Lycopersicon esculentum*) [8], and sweet potato (*Ipomoea batatas* L.) [9]; two *CAT* members (*CAT1* and *CAT2*) in barley (*Hordeum vulgare* L.) [10]; three *CAT* members (*CAT1–CAT3*) in tobacco (*Nicotiana plumbaginifolia*) [11], maize (*Zea mays* L.) [12], pumpkin (*Cucurbita* Linn.) [13], *A. thaliana* [14], and rice (*Oryza sativa* L.) [15]; and four (*CAT1–CAT4*) in cucumber (*Cucumis sativus* L.) [16] and rice [17]. So far, the highest number (7) of *CAT* genes has been documented in cotton (*Gossypium hirsutum* L. and *G. barbadense* L.) [18].

Several investigations have recommended that *CAT* genes’ expression is modulated temporally and spatially and reacts to numerous environmental pressures [9,19,20,21,22,23]. In *A. thaliana*, *CAT1* is a vital gene that responds to numerous abiotic stresses by scavenging H_2_O_2_. Moreover, *CAT2* and *CAT3* are involved in eliminating H_2_O_2_ subsidized to ROS homeostasis in light or dark conditions [4]. According to Du et al. [14], *CAT2* could be triggered in response to drought and cold stresses, while *CAT3* was primarily triggered by abscisic acid (ABA) and oxidative treatments at the senescence phase. In *A. thaliana*, it has been validated that *CAT2* is the main isoform in the leaf, which is systematically connected with photorespiration [24]. Additionally, Contento and Bassham [25] reported that *A. thaliana cat2* mutants show areas of chlorosis and necrotic scratches. In *Ipomoea batatas*, the transcript level of *CAT1* was persuaded via ethephon and stimulated by condensed glutathione (GSH), nicotinamide adenine dinucleotide phosphate (NADPH) oxidase inhibitor diphenylene iodonium (DPI), calcium ion (Ca^2+^) chelator egtazic acid, and cycloheximide [9]. Moreover, transgenic *N. tabacum* plants carrying the *CAT2* gene from maize showed improved tolerance to pathogen contagion and oxidative stress [26].

Likewise, the expression levels of the *CAT* genes vary in different tissues. For instance, in hot pepper (*Capsicum annuum* L.), the transcript levels of *CAT1*–*CAT3* in diverse organs (i.e., leaf and stem) are linked to circadian rhythms and different stresses [27]. In tobacco, *CAT1* and *CAT2* are spotted in non-senescent leaves; the transcript pattern of *CAT2* is expressively decreased in senescent leaves. In contrast, *CAT3* is spotted in both types of plants [28]. In Scots pine (*Pinus sylvestris* L.), *CAT* is elaborated in embryogenesis and cell death procedures [29]. In cucumber, *CAT1–CAT3* express in different organs (root, stem, leaves, flowers, and fruits), and could be significantly induced by salinity, cold, drought, and ABA treatments [16].

Moreover, previous investigations advised that miRNA-mediated modulation of ROS-associated genes is imperative for plant growth and development [30,31], as well as for stress resistance [32,33,34,35]. In Chinese cottonwood (*Populus simonii*), the interaction network between DNA methylation and miRNA (Ptc-miR396s and Ptc-miR156s)-target genes, and their products, affect the metabolic aspects of the antioxidant-encoding genes such as *SOD* and *CAT* under temperature stress. Results show that DNA methylation possibly modulates the transcript levels of the miRNA genes, therefore distressing the transcript of their target genes [36]. Likewise, Wang et al. [18] prophesied the miRNA-facilitated modulation of cotton *CAT* genes and detected some presumed target positions (catalase domain) of cotton miRNAs (novel_mir_2537/0819, miRCS46a, miRCS46a/gb-miR7486, and ghr-miR6138, etc.) under *Verticillium dahlia* infection. These findings specified that miRNAs might play vital roles in response to environmental cues via modulating the CAT genes.

Until now, *CAT* genes have been documented in several plant species and investigated under different stress conditions. Particularly, the *CAT* gene family has not been reported in rapeseed. Rapeseed (*Brassica napus* L.) is considered the second major oilseed crop after soybean (*Glycine max* L.). It has a composite genome because of its evolutionary past. Rapeseed yield restrictions have been changed owing to the augmented experience of multiple abiotic stresses [37,38,39]. Therefore, a genome-wide comprehensive investigation has been carried out to identify the *CAT* gene family in rapeseed for the first time. Further, their phylogenetic relationships, synteny analysis, gene structures, conserved motifs, *cis*-elements, miRNA regulator prediction, and functional annotation have been characterized. Furthermore, the expression pattern in different tissues and under various hormone and abiotic stresses has been widely evaluated to get insight into the rapeseed *CAT* genes.

## 2. Results

### 2.1. Identification of CAT Genes Family in Rapeseed

In the present study, a total of 14 *CAT* genes were recognized in the complete genome of rapeseed via BLASTP using 3 AtCAT protein sequences as queries (Table 1). These genes were named *BnCAT1–BnCAT14,* among which seven genes (*BnCAT1–BnCAT7*) were positioned in the A subgenome, and five genes (*BnCAT8–BnCAT12*) were positioned in the C subgenome (Table 1). *BnCAT13* and *BnCAT14* are distributed in two scaffold regions (Bnascaffold0025 and Bnascaffold0025) and have not yet been incorporated into the physical map of chromosomes (Table 1). Detailed information of the 14 *BnCAT* genes is presented in Table 1. Briefly, gene length ranged from 1990 bp (*BnCAT6*) to 9770 bp (*BnCAT10*) with four to five exons in each sequence. Notably, *BnCAT5* comprises six exons, and three genes (*BnCAT3, BnCAT9,* and *BnCAT10*) consisted of 12 and 15 exons, respectively. The coding DNA sequences (CDS) ranged from 1434 bp (*BnCAT13*) to 3123 bp (*BnCAT10*), while the protein length varied from 477 (*BnCAT13*) to 1040 (*BnCAT10*) amino acids. The prophesied molecular weights of the 14 BnCAT proteins ranged from 55.19 kDa (*BnCAT13*) to 565.40 kDa (*BnCAT6*), and the isoelectric points extended from 6.5 (*BnCAT1*) to 8.42 (*BnCAT5*). The subcellular localization outcomes predicted that nine BnCAT proteins were positioned on the peroxisome, while the remaining proteins were located on cytoskeleton (BnCAT1), cytoplasm (BnCAT4), mitochondrion (BnCAT9), and chloroplast (BnCAT5 and BnCAT10) (Table 1). Meanwhile, 4 and 7 *CAT* genes were also identified from the *Brassica oleracea (BoCAT1-BoCAT4)* and *Brassica rapa (BraCAT1-BraCAT7)* genomes, respectively (Table S2).

### 2.2. Phylogenetic Relationships of CAT Genes

**Figure 1:**
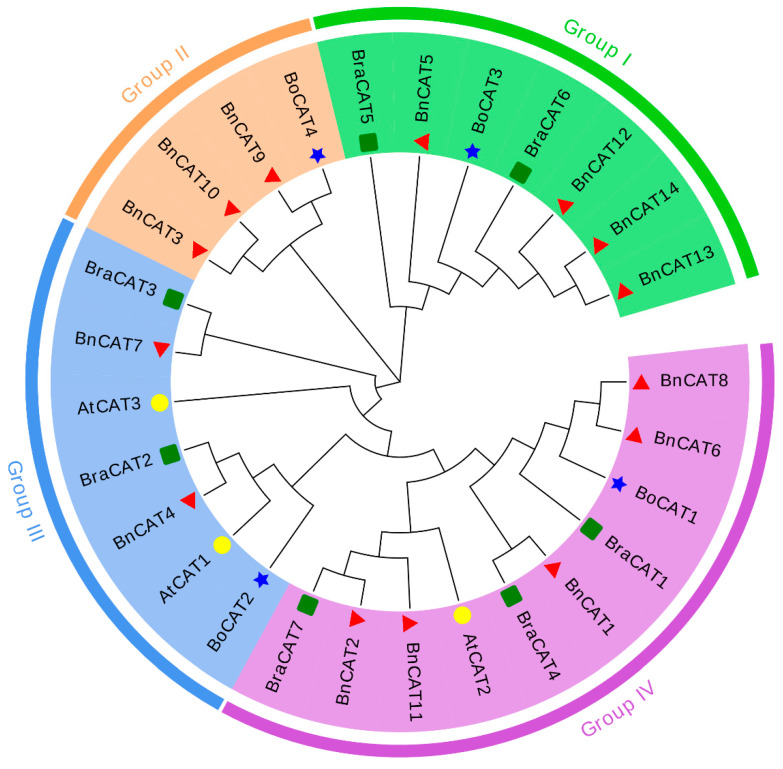
A neighbor-joining phylogenetic tree analysis of CAT proteins in *Brassica napus, Brassica oleracea, Brassica rapa,* and *Arabidopsis thaliana*. Overall, 14 *BnCATs* from *Brassica napus* (red triangle), 4 *BoCATs* from *Brassica oleracea* (blue star), 7 *BraCATs* from *Brassica rapa* (green box), and 3 *AtCATs* from *Arabidopsis thaliana* (yellow circles) were clustered into four groups (Groups I–IV), represented by different colors.

To explore the evolutionary relationships between the *BnCAT*, *BoCAT, BraCAT*, and *AtCAT* genes, an unrooted phylogenetic tree was constructed, which was clustered into four groups (Groups I–IV) (Figure 1). Our results showed that Group I comprised of 7 *CAT* members (4 *BnCATs*, 2 *BraCATs*, and 1 *BoCAT*); Group II comprised of 4 *CAT* members (3 *BnCATs* and 1 *BoCAT*); Group III comprised of 7 *CAT* members (2 *BnCATs*, 2 *BraCATs*, 1 *BoCAT*, and 2 *AtCATs*); and Group IV comprised of 10 *CAT* members (5 *BnCATs*, 3 *BraCATs*, 1 *BoCAT*, and 1 *AtCAT*). Notably, the *BnCAT* genes were distributed in four groups, and Group IV was found to contain more *BnCATs* than the other three groups (Figure 1). Furthermore, it was found that the *BnCATs* have a closer phylogenetic relationship with the *BoCATs* and *BraCATs* in each group.

### 2.3. Synteny Analysis of CAT Genes

**Figure 2:**
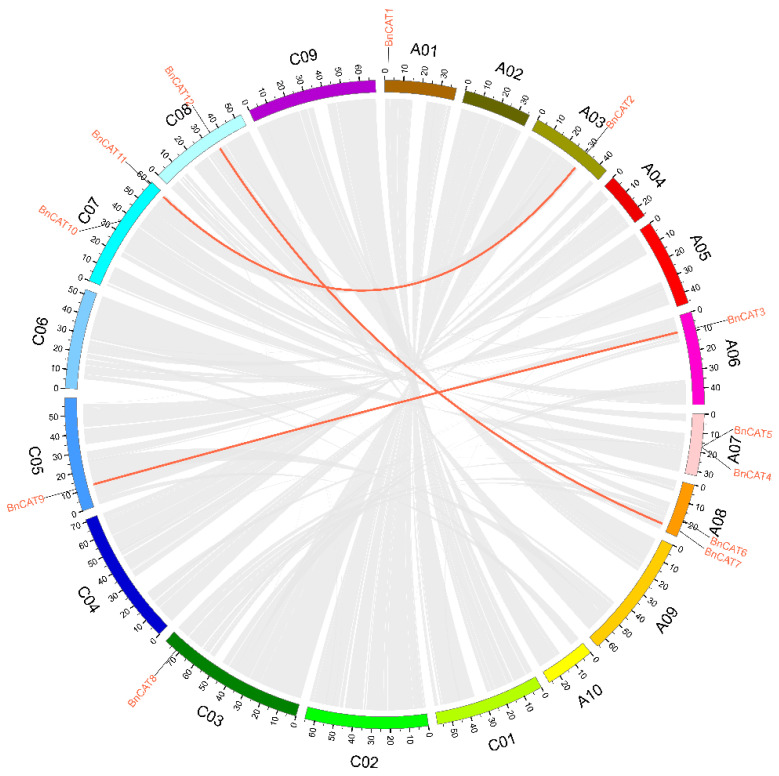
Circular illustrations of the chromosomal dispersal and inter-chromosomal associations of *BnCAT* genes. Grey lines in the background show all the syntenic blocks in the rapeseed genome, and the red lines show syntenic CAT gene pairs.

Tandem and segmental duplication promote new gene family members’ development and plant genome progression. The segmental and tandem duplication actions in the *BnCAT* gene family were examined to clarify the rapeseed *BnCAT* gene duplication actions. The chromosomal dispersals of 12 *BnCAT* genes were evaluated. According to Figure 2, 9 out of the 19 chromosomes possessed *BnCAT* genes. Briefly, chromosomes A07, A08, and C07 have two *BnCAT* genes, whereas chromosomes A01, A03, A06, C03, C05, and C08 possessed only one *BnCAT* gene (Figure 2). Notably, the remaining chromosomes did not contain any *BnCAT* gene. Regardless of the chromosomes, A01, A07, and C03 have one and two genes, and no tandem repeat paralogous genes were found in these regions. In addition, six paralogous genes were recognized on the A03, A06, A08, C05, C07, and C08 chromosomes (Figure 2). One tandem duplication on chromosome A07, one proximal duplication on chromosome A08, and 6 duplication gene pairs were detected (Figure 2). These outcomes showed that the duplication actions played a crucial role in expanding the *BnCAT* family genes.

**Figure 3:**
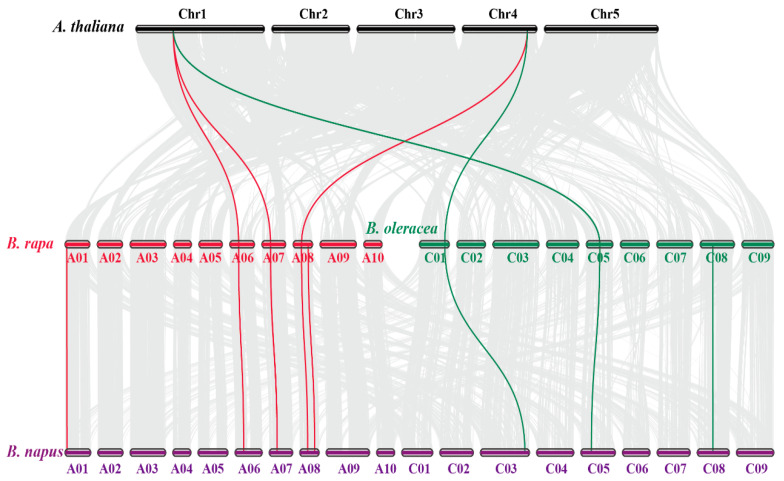
Synteny analysis of *CAT* genes in *B. napus, B. rapa, B. oleracea,* and *A. thaliana* chromosomes. Grey lines in the background show the collinear blocks within rapeseed and other plant genomes, while the red and green lines highlight the syntenic *CAT* gene pairs. Genes positioned on the *B. napus* A genome are syntenic with *B. rapa* and *A. thaliana*, while genes positioned on the *B. napus* C genome are syntenic with *B. oleracea* and *A. thaliana*.

Collinearity analysis discovered robust orthologs of the *CAT* genes among *B*. *napus* and the other three inherited plant species (Figure 3; Table S3). Briefly, in the A subgenome, 4 *B. napus* genes displayed syntenic associations with 3 *AtCATs* and 1 *BraCAT*. On the other hand, in the C subgenome, 3 *B. napus* genes showed syntenic associations with 2 *AtCATs* and 1 *BoCAT* (Figure 3). Notably, several homologous of *A. thaliana*, *B*. *rapa,* and *B*. *oleracea* sustained a syntenic association with *BnCATs*, suggesting that whole-genome duplication played a key role in *BnCATs* gene family evolution along with segmental repetition (Table S3).

To comprehend the evolutionary constrictions on the *BnCAT* gene family, the Ka, Ks, and Ka/Ks ratio was assessed for rapeseed. Our findings presented that all the duplicated *BnCAT* gene pairs had a Ka/Ks ratio of <1 (Table S3), demonstrating that the rapeseed *CAT* family genes might have faced selective pressure, a discriminating burden, throughout its evolution.

### 2.4. Analysis of BnCAT Gene Structures and Conserved Motifs

**Figure 4:**
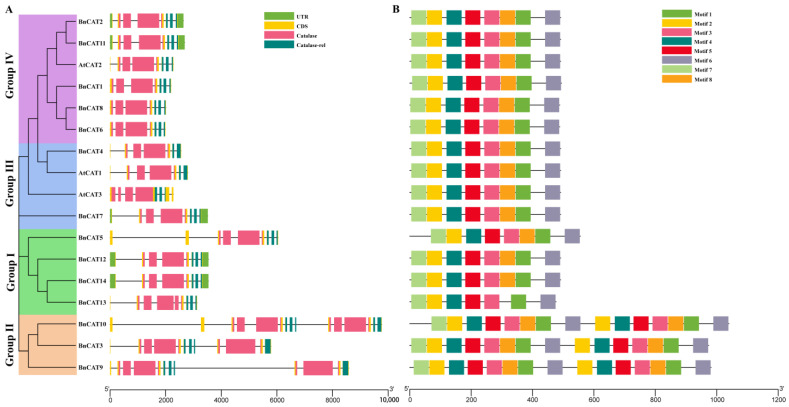
The gene structure and motif analysis of the *CAT* family genes from *B. napus* and *A. thaliana*. According to the phylogenetic relationships, the *CAT* genes from both genomes were clustered into four groups (Groups I–IV). (**A**) The gene structure of the *BnCATs* and *AtCATs*. Light green color shows the UTR regions, yellow color shows the CDS or exons, black horizontal line shows the introns, pink color shows the catalase core domain (catalase, PF00199), and dark green color shows the catalase-related immune-responsive domain (catalase-rel, PF06628). (**B**) Conserved domain structures identified in the *BnCATs* and *AtCATs*. Different color boxes show different motifs.

The exon–intron configurations of the *BnCAT* genes were observed to boost our understanding of the rapeseed *CAT* family genes’ development. We found that introns of the *BnCATs* ranged from 6 to 14 (Figure 4A). Groups III and IV contained 6 to 7 introns, Group I includes 7 to 8 introns, whereas Group I had the highest number of introns (11 and 14). Similarly, Groups I, III, and IV contain 4 to 6 exons, while Group I had the highest number of exons (12 and 15). Some of the exons are overlapped with the catalase-domain regions (Table 1; Figure 4A). Briefly, Groups I, III, and IV showed similar intron/exon association patterns, and only Group I had a diverse intron/exon association pattern. These findings specified that members inside a group had remarkably identical gene structures, consistent with their phylogenetic associations.

Moreover, we investigated the full-length protein sequences of 14 *BnCATs* and 3 *AtCATs* via MEME software to recognize their conserved motifs. The conserved motif of the *CAT* genes ranged from 8 to 15. Overall, 8 conserved motifs were identified, and the motif distributions were also similar within the groups (Groups I–IV) (Figure 4B; Table S4). For instance, Groups I, III, and IV contain 8 conserved motifs, except *BnCAT13*. Moreover, all members of Group II contained 15 conserved motifs (Figure 4B). In conclusion, the group classifications’ consistency was powerfully maintained by analyzing the conserved motifs’ composition, gene structures, and phylogenetic relationships, signifying that the CAT proteins have extremely preserved amino acid remains, and members inside a group may have parallel roles.

### 2.5. Cis-Elements in Promoters of BnCAT Genes

**Figure 5:**
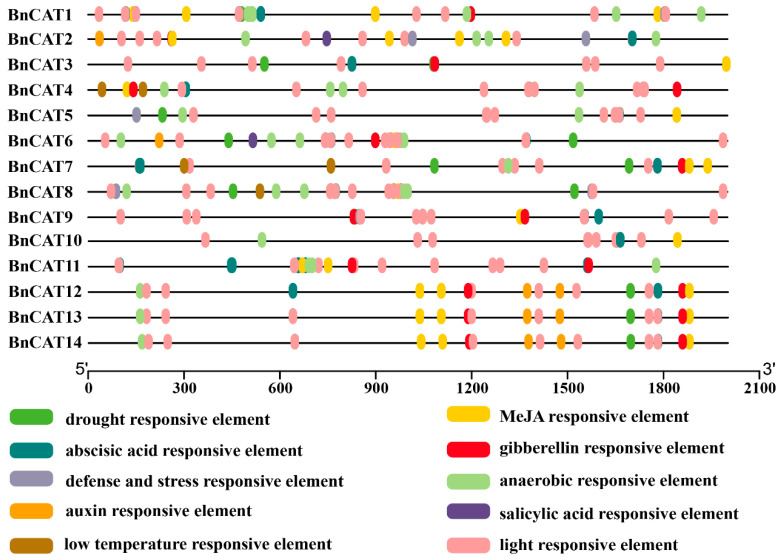
*Cis*-elements in the promoters of the *BnCAT* genes that are associated with different hormone- and stress-responsive elements. Different color boxes show different identified *cis*-elements.

To recognize the gene functions and regulatory patterns, the *cis*-elements in the promoters of the *BnCAT* genes were investigated by searching a 2000 base-pair (bp) region from the transcriptional activation site of each gene against the PlantCARE database. As shown in Figure 5, several *cis*-regulatory elements were predicted in the *BnCAT* promoter. The detailed information of the identified elements is presented in Table S5. Overall, five different hormone-correlated responsive elements related to abscisic acid (ABA), auxin, methyl jasmonate (MeJA), gibberellin (GA), and salicylic acid (SA) were identified, suggesting that these genes may respond to phytohormones (Figure 5; Table S5). Most of the hormone-correlated responsive elements are very specific to some genes, as shown in Figure 5. Among them, the ABA-, MeJA-, and GA-responsive elements are widely distributed and commonly present in several genes, highlighting their vital roles in phytohormone responses (Figure 5).

Additionally, four different stress-related responsive elements (drought, low-temperature, anaerobic, and light) were also predicted, indicating that these genes may respond to stress stimulates (Figure 5; Table S5). Moreover, a number of light-responsive elements were observed, suggesting the significant role of *BnCATs* in response to light stress. Besides, one defense and stress-responsive element were identified in *BnCAT1, BnCAT2, BnCAT5, BnCAT8,* and *BnCAT9* genes (Figure 5; Table S5), demonstrating that these five genes might participate against defense and stress. These hormone and stress-associated elements in the *BnCAT* promoters suggest that transcript profiling of *BnCATs* may vary with hormones and abiotic stresses.

### 2.6. Genome-Wide Analysis of miRNA Targeting BnCAT Genes

**Figure 6:**
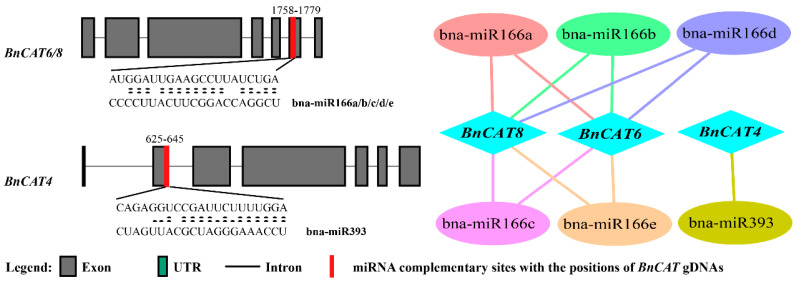
A network illustration of the regulatory associations among the presumed miRNAs and selective *BnCAT* genes.

Over the past few years, a significant number of investigations have revealed that miRNA-mediated regulation is linked with the stress response in plants. Therefore, to boost our understanding of the miRNAs associated with the regulation of *BnCAT* genes, we identified 6 putative miRNAs targeting 3 *BnCAT* genes, as shown in the network diagram (Figure 6). The detailed information of the miRNA targeted sites is presented in Table S6. We found that five members of the bna-miR166 family targeted two genes (*BnCAT6* and *BnCAT8*), and one member of the bna-miR393 family targeted one gene (*BnCAT4*) (Figure 6; Table S6). Particularly, *BnCAT6* and *BnCAT8* were predicted to be targeted by five miRNAs (bna-miR166a, bna-miR166b, bna-miR166c, bna-miR166d, and bna-miR166e).

### 2.7. Functional Annotation Analysis of BnCAT Genes

To further recognize the *BnCAT* genes’ functions, we performed GO annotation and enrichment analysis based on biological process (BP), molecular function (MF), and cellular component (CC) classes; these terms help us understand the function of genes at a molecular level. The annotation results of BP, MF, and CC exhibited several significantly enriched terms (Table S7). Some of the most enriched terms are highlighted in the subsequent section.

The GO-CC enrichment analysis confirmed that 14 terms are highly enriched, such as peroxisome (GO:0005777), cytoplasmic part (GO:0044444), cellular_component (GO:0005575), organelle (GO:0043226), cytoplasm (GO:0005737), mitochondrion (GO:0005739), and cell (GO:0005623), etc. (Table S7). Some of these terms are consistent with the subcellular localization prediction of the CAT proteins. The GO-MF enrichment results detected 13 highly enriched terms, including catalase activity (GO:0004096), organic cyclic compound binding (GO:0097159), oxidoreductase activity, acting on peroxide as acceptor (GO:0016684), catalytic activity (GO:0003824), antioxidant activity (GO:0016209), and peroxidase activity (GO:0004601), etc. (Table S7). These terms also confirm the function of *BnCAT* genes in ROS scavenging and antioxidant defense systems. Likewise, the GO-BP results predicted a vast number (33) of significantly enriched terms. The most common and useful terms are a cellular response to stimulus (GO:0051716), ROS metabolic process (GO:0072593), response to stress (GO:0006950), response to ROS (GO:0000302), response to hydrogen peroxide (GO:0042542), and cellular detoxification (GO:1990748), etc. (Table S7). Overall, GO enrichment analysis confirms the functional role of *BnCAT* genes in several cellular, molecular, and biological processes related to ROS, antioxidant enzymes, and response to stresses.

### 2.8. Expression Profiling of BnCAT Genes in Various Tissues

**Figure 7:**
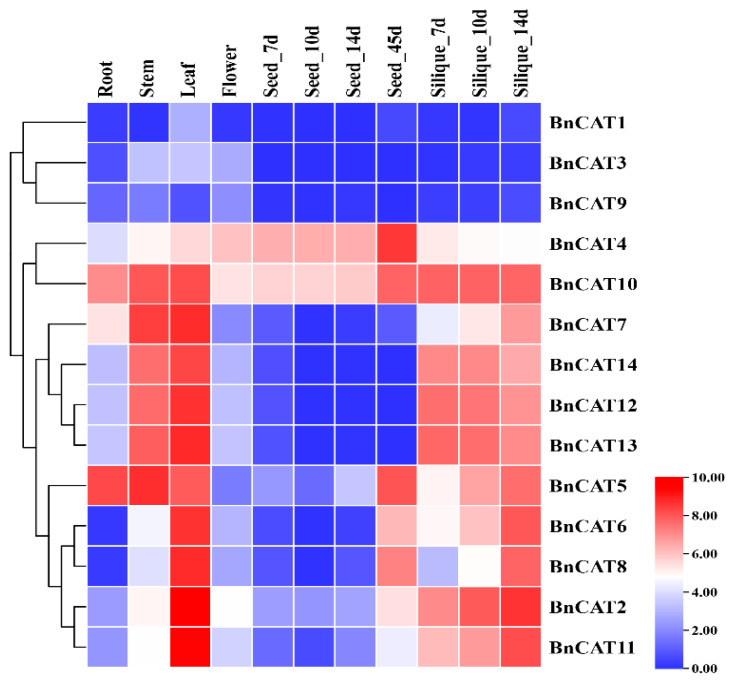
Expression patterns of *BnCAT* genes in various tissues at different growth phases of rapeseed. The 7d, 10d, 14d, and 45d labels showed the time-points when the samples were harvested. In the expression bar, the red color shows high, and the blue color shows low expression levels.

To illustrate the transcript levels of the *BnCAT* genes, we examined 11 tissues and organs of rapeseed at various growth phases based on RNA-seq data from rapeseed (ZS11 variety) (BioProject ID PRJCA001495). The expression profiles of the *BnCAT* genes varied in the various tissues and organs. For instance, the expression patterns of most *BnCAT* genes in stem, leaf, and silique-5d, silique-7d, silique-10d, and silique-14d were higher than those of other tissues (Figure 7). The expression levels of *BnCAT1* (TPM = 0.00–6.44), *BnCAT3* (TPM = 0.00–9.91), and *BnCAT9* (TPM = 0.01–3.45) were much lower, respectively; in contrast, *BnCAT4* (TPM = 14.98–374.72) and *BnCAT10* (TPM = 40.89–287.88) were highly expressed in most of the tissues, respectively, suggesting that these candidate genes may play diverse roles in regulating rapeseed growth processes (Figure 7).

### 2.9. Expression Profiles of BnCAT Genes under Phytohormones and Abiotic Stress Conditions

**Figure 8:**
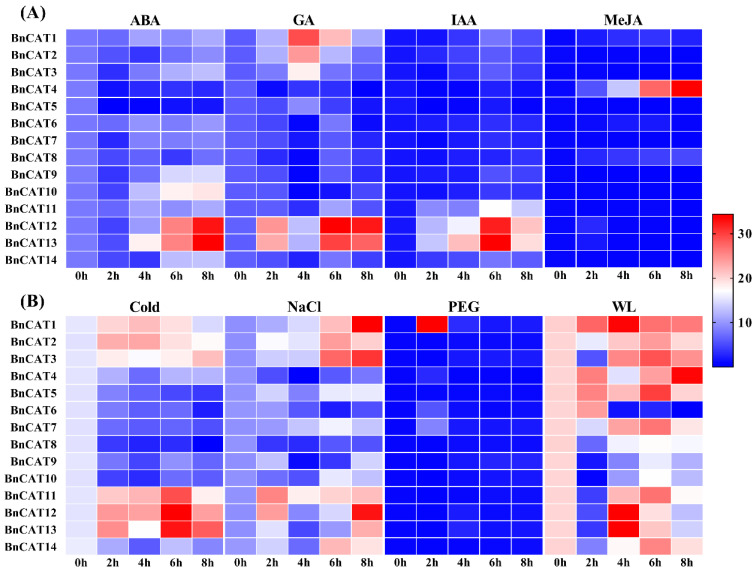
Expression patterns of the *BnCAT* genes under various hormones and abiotic stress. (**A**) Expression profiling during hormone treatments, including abscisic acid (ABA), gibberellic acid (GA), indole-acetic acid/auxin (IAA), and methyl jasmonate (MeJA). (**B**) Expression profiling under abiotic stresses, including cold, salinity (NaCl), drought (PEG-6000), and waterlogging (WL). The 0 h (CK), 2 h, 4 h, 6 h, and 8 h labels displayed the time points (hours) when the samples were harvested for expression analysis after the stress treatment. The bars show the comparative gene expression patterns examined via the 2^−^^ΔΔ^^CT^ method. The red color shows high, and the blue color shows low expression levels.

Under abiotic and phytohormone stress conditions, rapeseed growth and development were affected by physiological, biochemical, and molecular levels. Therefore, qRT-PCR was applied to investigate 14 *BnCAT* genes’ expression levels at different time points after drought, salinity, cold, waterlogging, ABA, GA, MeJA, and IAA treatments (Figure 8). Under hormone stress conditions, most of the genes showed relatively low expression levels, except for some genes. For instance, *BnCAT12* in response to ABA (2.73 and 3.40 fold), GA (3.67 and 3.48 fold), and IAA (8.85 and 6.08 fold), as well as *BnCAT13* in response to ABA (2.72 and 3.58 fold), GA (3.24 and 3.04 fold), and IAA (9.47 and 5.61 fold) at 6 and 8 h were significantly upregulated, respectively. Further, *BnCAT10* (1.99–2.08 fold) display substantially higher expression at 6 and 8 h under ABA treatment, respectively (Figure 8A). Under GA treatment, *BnCAT1* (2.48–3.17 fold) at 4 and 6 h, and *BnCAT2* (2.71 fold) at 4 h were expressively upregulated, respectively (Figure 8A). Only *BnCAT4* (27.49–34.37 fold) was upregulated after 6 and 8 h of the MeJA treatment, respectively (Figure 8A). However, *BnCAT5–BnCAT10* and *BnCAT14* genes did not display any significant difference throughout the hormone treatments (Figure 8A).

Meanwhile, a relatively high number of *BnCAT* genes were upregulated by abiotic stresses compared to hormone treatments concerning their control conditions (Figure 8B). Specifically, *BnCAT1–BnCAT3* (1.33–1.41 fold) and *BnCAT11–BnCAT13* (1.36–1.97 fold) were upregulated by cold stress at the early or late response, respectively (Figure 8B). Under salinity stress conditions, *BnCAT1–BnCAT3* (1.75–2.03 fold), *BnCAT11* (1.46–1.84 fold), and *BnCAT12* (1.25–2.21 fold) were upregulated at different time points of the treatment, respectively. Likewise, only *BnCAT1* (31.95 fold) was induced by drought stress at 2 h, and the other genes were not affected by the drought stress, respectively (Figure 8B). Interestingly, almost all of the genes were significantly upregulated at all time points under waterlogging stress, except *BnCAT8–BnCAT10* (0.14–0.87 fold), respectively, which showed relatively low expressions (Figure 8B). Briefly, some genes, such as *BnCAT1, BnCAT2, BnCAT3,BnCAT11, BnCAT12,* and *BnCAT13*, showed a similar expression pattern during ABA (0.93–1.33, 0.60–1.15, 0.56–1.47, 0.91–1.34, 0.69–3.40, and 0.75–3.58 fold), GA (1.47–3.17, 1.13–2.70, 0.99–2.14, 0.73–1.45, 1.63–3.67, and 1.55–3.24 fold), IAA (0.98–2.58, 1.30–2.12, 0.85–2.15, 2.76–5.02, 3.72–8.85, and 3.97–9.47 fold), cold (0.97–1.34, 1.12–1.41, 1.09–1.33, 1.16–1.71, 1.44–1.98, and 1.11–1.89 fold), and salinity (1.11–2.30, 1.30–1.75, 1.22–2.11, 1.47–1.84, 0.98–2.21, and 0.77–1.70 fold) treatments, respectively (Figure 8). The above outcomes suggest that these genes may be crucial for enlightening resistance to multiple stresses, especially waterlogging stress.

## 3. Discussion

### 3.1. Characterization of CAT Gene Family in Rapeseed

Rapeseed is an allotetraploid type that practiced extensive genome doubling and integration procedures [40]. In the current study, we recognized 14 *BnCAT* genes via genome-wide analysis that surpasses the 4 *BoCATs* and 7 *BraCATs* (Table 1; Table S2). Generally, the *CAT* gene family of plants usually consists of a small number of genes. Each *CAT* member in *A. thaliana* was naturally homologous to 2–3 genes in the rapeseed genome. These results are supported by the previous discovery that 1 *A. thaliana* gene paralleled to 2 or >2 homologous genes in rapeseed [41]. According to the phylogenetic investigation, 14 *BnCATs* were classified into four groups. *CAT* genes from *A. thaliana* were classified into the Group III and Group IV together with some *BnCATs* genes, *BoCATs,* and *BraCATs* (Figure 1), signifying comparable evolutionary lines among *B. napus*, *B. oleracea*, *B. rapa*, and *A. thaliana*. These alliances in the phylogenetic tree was further confirmed by the gene structure analysis (Figure 4). The locations and numbers of introns of the *CAT* genes were found to be extremely conserved between 13 angiosperm plants (monocots and dicots). This result suggests that one ancestral duplicate of the *CAT* gene, comprising seven introns, have gone over numerous rounds of intron loss and gain throughout evolution [42]. Moreover, the motif patterns were also comparable within the group classification (Figure 4B). Notably, Group II contains diverse motif patterns, suggesting that *BnCATs* possess exceptionally conserved protein structures (Figure 4B). These results are in agreement with previous findings of cotton, where genes within the same group contain diverse gene structures and motifs patterns [18].

### 3.2. The Critical Role of CAT Genes in ROS Metabolism, Hormone, and Abiotic Stress Responses

Catalase, a robust enzyme that facilitates the ROS-scavenging procedure, serves as a crucial player in plant growth, development, and abiotic stress response [2,3]. To date, it has been extensively studied in several plant species against various stresses, such as *A. thaliana* [43,44,45,46,47], rice [48,49,50], wheat [51,52,53], sugarcane [21,54,55], cucumber [16,56,57], cotton [18,58,59], and sweet potato [20,60,61].

Under drought and salinity stress, the *AtCAT1* transcript level was triggered by a MAPK kinase (*AtMEK1*), which was associated with ROS generation (mainly H_2_O_2_) [62]. The transcription factor WRKY75 suppressed the expression level of *AtCAT2* to improve the ROS accretion, leading to leaf senescence in *A. thaliana* [63]. In another study, the transcript level of *AtCAT2* was repressed to activate endogenous ROS generation under lead toxicity [64]. Further, Due et al. [14] discovered that *AtCAT1* played a significant role in eliminating abiotic stress-persuaded H_2_O_2_, whereas *AtCAT2* and *AtCAT3* ensured H_2_O_2_ scavenging under light and dark conditions, respectively. Additionally, *AtCAT3* was phosphorylated through a calcium-associated protein kinase to intercede H_2_O_2_ homeostasis inside the peroxisomes of guard cells under drought conditions, and the over-expression of *AtCAT3* improved the drought resistance in *A. thaliana* [43].

In rice, three *CAT* genes (*OsCatA, OsCatB*, and *OsCatC*) were triggered under different stress conditions; further, these genes were also involved in ROS homeostasis, root evolution stimulation, and photorespiration [15]. The over-expression of *OsCatA* and *OsCatC* improved the drought tolerance in transgenic rice [15]. In another study, Vighi et al. [65] reported that *OsCatA* and *OsCatB* were strongly induced by high salinity and cold stress in a tolerant genotype compared with that in a sensitive genotype. In cucumber, the expression level of *CsCAT1* was triggered by ABA treatment, whereas *CsCAT2* was repressed under drought stress and *CsCAT3* upregulated after salinity, drought, and ABA treatment [16]. In sugarcane, the expression pattern of *ScCAT1* was induced in response to oxidative, salinity, heavy metal, and drought stresses [55]. In sweet potato, the transcript level of *IbCAT2* was stimulated under drought and salinity conditions, and the over-expression of this gene increases the tolerance to both stresses in *Saccharomyces cerevisiae* and *E. coli* [20].

In this study, *BnCAT1, BnCAT2, BnCAT3, BnCAT12,* and *BnCAT13* were mainly induced by ABA, GA, IAA, cold, and salinity. Interestingly, almost all identified genes were strongly influenced by waterlogging treatment, except *BnCAT8–BnCAT10*, which showed relatively low expressions throughout the stress; this indicates that these genes play a major role in waterlogging stress tolerance in rapeseed. Overall, these findings suggest that *CAT* genes play a significant part in activating ROS metabolism and respond to numerous abiotic cues (Figure 8). These results were also confirmed by the GO enrichment analysis, where most of the GO terms were found to be associated with ROS metabolism and abiotic stresses (Table S4). In future work, stress-related *CAT* genes can be used for functional validation in rapeseed to get further insight into their actual stress tolerance mechanisms.

### 3.3. Expression Pattern of CAT Genes in Various Tissues

The expression patterns of the *CAT* genes in different tissues have been described for numerous plant species. For example, in rice, *OsCATA* displayed a relatively higher expression level at all the developmental phases; *OsCATC* presented a nearly comparable expression pattern as *OsCATA*, apart from the flowering phase. *OsCATD* sustained a low constitutive expression level during the complete growth life cycle of rice [17]. In cotton, *GhCAT1–GhCAT4* significantly showed higher expression in almost all the growth and developmental stages (root, stem, leaf, petal, calycle, torus, stamen, pistil, and fiber development), suggesting that the *CAT* gene family is possibly associated with the growth and development of various tissues in cotton [18]. In cucumber, *CsCAT1* and *CsCAT2* showed higher transcript levels in leaves and considerable expression in fruit and roots, whereas *CsCAT2* showed a stronger transcript pattern in fruits, flowers, stem, and leaves [16]. In the current study, the results showed that many genes showed higher transcript levels in the root, stem, leaf, and silique (Figure 7). Only *BnCAT4* (TPM = 82.23–374.72) and *BnCAT10* (TPM = 49.85–219.51) exhibited higher expression throughout the seed expansion stages. The expression levels of 3 genes (*BnCAT1* (TPM = 0.00–6.44)*, BnCAT3* (TPM = 0.00–9.91), and *BnCAT9* (TPM = 0.01–3.45) in all the tissues were almost undetectable in rapeseed, indicating that these genes might do not play any role in rapeseed growth and development (Figure 7).

### 3.4. miRNA: Key Players in the Regulation of Stress Responses

MicroRNAs (miRNAs), a group of single-stranded, non-coding micro RNAs, have been revealed to be involved in post-transcriptional gene regulation [30,66]. During the past few years, numerous miRNAs have been identified via genome-wide analysis in rapeseed that responds to different environmental stresses [67,68,69,70,71,72,73].

The current study recognized five members of the bna-miR166 family and one member of the bna-miR393 family targeting three *CAT* genes (*BnCAT4, BnCAT6,* and *BnCAT8*) (Figure 6). miR166 has been reported to be significantly upregulated in response to UV-B radiation in maize [74]; in cassava under cold and drought stresses conditions [75]; and in Chinese cabbage under heat stress conditions [75]. Likewise, miR393 has also been reported to be strongly linked with stress responses, such as drought and salinity in French bean (*Phaseolus vulgaris*) [76], cold stress in tea plant (*Camellia sinensis*) [77], and nitrogen deficiency in maize [78]. Some of these miRNAs have also been reported in rapeseed, playing a significant role in rapeseed genetic improvement [67,68,69,70,71,72,73]. These studies suggest that these bna-miRNAs might play decisive roles against numerous stresses by modifying the transcript level of the *CAT* genes in rapeseed.

## 4. Materials and Methods

### 4.1. Identification of CAT Family Genes

In this work, two approaches were used to identify the *CAT* genes in rapeseed. Firstly, the rapeseed genome (ZS11 genotype) sequence was downloaded from the BnPIR database (http://cbi.hzau.edu.cn/bnapus/index.php, accessed on 10 February 2021), and a local rapeseed dataset was used by software blast-2.7.1+ (ftp://ftp.ncbi.nlm.nih.gov/blast/executables/blast+/2.7.1/, accessed on 10 February 2021). The amino acid sequences of 3 *A. thaliana CAT* family genes (*AtCAT1*/At1g20630, *AtCAT2*/At4g35090, and *AtCAT3*/At1g20620) were obtained from the TAIR *Arabidopsis* genome database (http://www.arabidopsis.org/, accessed on 10 February 2021). These sequences were used as a reference to identify the *CAT* genes in the local rapeseed database.

Secondly, the HMMER 3.1 (http://www.hmmer.org/, accessed on 10 February 2021) and BLASTP with the threshold e-value set to 1e^−5^ were performed using the Hidden Markov Model (HMM V 3.0) profiles of the catalase (PF00199) and the catalase-related immune-responsive domain (PF06628) were downloaded from the Pfam protein database (http://pfam.xfam.org/, accessed on 10 February 2021) and used as the inquiry. The default limitation of HMMER 3.1 was set to 0.01. We combined all hits based on HMMER and BLASTP results and omitted redundant hits with a similar scaffold or chromosome location due to the rapeseed genome’s complexity. The remaining *CAT* sequences were scrutinized for a catalase-related immune-responsive domain (PF06628) by the Pfam server to confirm that all the identified genes belonged to the *CAT* gene family. Finally, 14 *BnCAT* genes were identified in the rapeseed genome. The genomic and protein sequences of all the identified *BnCAT* genes were obtained from the BnPIR rapeseed database. Moreover, to disclose the *CAT* genes’ evolutionary association in different plant species, the protein sequences of the *Brassica rapa* and *Brassica oleracea* CATs were downloaded from the JGI Phytozome 12.0 (https://phytozome.jgi.doe.gov/pz/portal.html, accessed on 10 February 2021) database via the same method as described above.

### 4.2. Characterization of CAT Family Genes

The physical and chemical properties, such as the number of amino acids in a sequence, molecular weight (MW), and isoelectric points (pI), were measured using the PROTPARAM tool on the ExPASy website (http://web.expasy.org/protparam/, accessed on 10 February 2021). The subcellular localization of BnCAT proteins was predicted using the WoLF PSORT server (https://wolfpsort.hgc.jp/, accessed on 10 February 2021). The structure of the *BnCAT* genes was predicted and constructed via TBtools software (V 1.068; https://github.com/CJ-Chen/TBtools, accessed on 10 February 2021). Potentially conserved motifs were identified using MEME (V 4.11.4) by analyzing the sequences of 13 BnCAT proteins.

### 4.3. Phylogenetic Tree and Synteny Analysis of BnCAT Family Proteins

To observe the evolutionary past of the rapeseed *CAT* gene family, we used *Brassica napus, Brassica oleracea, Brassica rapa,* and *A. thaliana* protein sequences to create a phylogenetic tree. The protein sequences were multiple-aligned using MEGA X (V 6.06) software. The tree was constructed based on the neighbor-joining (NJ) method with 1000 bootstrap replicates. For better visualization, the tree was observed and modified via the online website Evolview v3 (https://www.evolgenius.info/evolview, accessed on 10 February 2021). Synteny relationships of *CAT* genes from *Brassica napus, Brassica oleracea, Brassica rapa,* and *Arabidopsis thaliana* were developed from JCVI (https://github.com/tanghaibao/jcvi, accessed on 10 February 2021). To investigate the evolutionary constrictions of each *CAT* gene pairs, the synonymous (Ks), non-synonymous (Ka) substitution, and Ka/Ks ratios were calculated using KaKs_Calculator 2.0 (https://sourceforge.net/projects/kakscalculator2/, accessed on 10 February 2021).

### 4.4. Cis-Elements Analysis in the BnCAT Gene Promoters

To classify the putative *cis*-elements, the 2000 base pairs (bp) upstream of the start codons were downloaded from the rapeseed genome database (BnPIR) for promoter analysis. Then, each promotor’s *cis*-elements were prophesied using the PlantCARE webtool (http://bioinformatics.psb.ugent.be/webtools/plantcare/html/, accessed on 10 February 2021) and were presented using TBtools (V 1.068).

### 4.5. Prediction of Putative miRNA Targeting BnCAT Genes and Functional Annotation Analysis

The gene sequences of the *BnCATs* were acquiesced as the candidate genes to identify possible miRNAs via observing against the existing rapeseed reference of miRNA sequences via the psRNATarget database (http://plantgrn.noble.org/psRNATarget/, accessed on 10 February 2021) with default parameters. Cytoscape (V3.8.2, https://cytoscape.org/download.html, accessed on 10 February 2021) software was used to create the interaction network between the prophesied miRNAs and the equivalent target *BnCAT* genes.

To predict the functional annotation of *BnCAT* genes, we performed the gene ontology (GO) annotation analysis by submitting all *BnCAT* gene sequences to the eggNOG database (http://eggnog-mapper.embl.de/, accessed on 10 February 2021). Then, the GO annotation data was handled in TBtools for GO enrichment analysis.

### 4.6. Expression Analysis of BnCAT Genes in Different Tissues

The temporal and spatial expression patterns of the candidate *BnCAT* gene family were evaluated using the RNA-seq data (BioProject ID: PRJCA001495). We examined the total RNA-seq data of the root, stem, leaf, flower, seed, and siliques at the germination stages of the rapeseed variety “ZS11”. Raw reads were quality controlled and filtered by software fastp (https://github.com/OpenGene/fastp, accessed on 10 February 2021), and then mapped to the reference genome with software HISAT2 (https://github.com/infphilo/hisat2, accessed on 10 February 2021). Sequence alignment map (SAM) files were converted to binary alignment map (BAM) and sorted with software Samtools (http://www.htslib.org/, accessed on 10 February 2021). Cuffquant and Cuffnorm were used to generate normalized counts in transcripts per million (TPM) values. Based on TPM values, the expression heat map was created using GraphPad Prism 9.0.0 software (https://www.graphpad.com/, accessed on 10 February 2021).

### 4.7. Plant Material and Stress Conditions

In this study, the rapeseed genotype “ZS11”, a typical cultivated variety, was used for stress treatments. The seeds of ZS11 genotype was furnished by OCRI, CAAS, China. Before stress treatments, some seeds were randomly selected from the same batch of seeds to determine the germination rate. The seeds with a 100% germination rate were considered vigorous seeds. The vigorous seeds were carefully chosen and sterilized with 10% hypochlorous acid solution for 5 min. The seeds were grown on water-saturated filter paper in a chamber (25 °C day/night and 16h/8h light/dark cycle) until the radicle’s extent reached about 5 mm. For stress treatment, germinated seeds were exposed to 150 mM NaCl solution for salinity stress, 15% PEG6000 solution for drought stress, and 4 °C for cold stress on water-saturated filter paper. To analyze the effect of different phytohormones, the germinated seeds were cultivated in Murashige and Skoog (MS) medium provided with 100 μM abscisic acid (ABA), 100 μM gibberellic acid (GA), 100 μM methyl jasmonate (MeJA), and 100 μM indole-acetic acid/auxin (IAA). The samples were collected at 0 (CK), 2, 4, 6, and 8 h after the treatments. Three biological replications were carried out for all the treatments. All the samples were instantly frozen in liquid nitrogen and were stored at −80 °C for the next analysis.

### 4.8. RNA Extraction and qRT-PCR Analysis

Total RNA was extracted using TransZol Up Plus RNA Kit (TransGen Biotech, Beijing, China) according to the manufacturer’s instructions and was then quantified with a Nanodrop ND-1000 spectrophotometer (Thermo Fisher Scientific, Worcester, MA, USA). RNA was purified using TransScript One-Step gDNA Removal. The first-strand of complementary DNA (cDNA) was synthesized using a cDNA Synthesis SuperMix kit (TransGen Biotech, China), and the cDNA solution was then diluted (20x) with distilled deionized water. The quantitative real-time polymerase chain reaction (qRT-PCR) was carried out with an ABI StepOne real-time fluorescence quantitative PCR instrument (Applied Biosystems, Foster City, CA, USA) using SYBR^®^ Green Supermix (Bio-Rad). The *BnACTIN* was used as an internal control. The qRT-PCR reaction was performed as follows: 94 °C for 10 min, followed by 40 cycles of 94 °C for 15 s, 60 °C for 30 s, and 72 °C for 10 s. Each qRT-PCR reaction was carried out with three biological triplicates, and the data were examined using the2^−ΔΔCT^ method as described by Wang et al. [79]. All the primers used in this experiment are listed in Table S1. The heat map was created using GraphPad Prism 9.0.0 software.

## 5. Conclusions

This study conducted a genome-wide comprehensive study of the *CAT* gene family in rapeseed and identified 14 *BnCAT* genes, which were clustered into four groups (Group I–Group IV). To get further insights, gene structure, conserved motifs, *cis*-elements, GO annotation, and miRNA prediction analysis have also been performed. Additionally, several genes’ expression levels were highly expressed in the root, leaf, stem, silique, and late stages of seed development. Likewise, several genes were mainly upregulated in response to the ABA, GA, IAA, cold, drought, and waterlogging treatments. Some of the genes are expressed at specific tissues and stress conditions. Briefly, the wide-ranging information collected in the current study can be exploited for future functional analysis of the *BnCAT* genes regarding rapeseed growth, development, response to hormones, and abiotic stresses.

## Figures and Tables

**Table 1:** Information of the 14 *BnCAT* genes identified in rapeseed.

Gene ID	Gene Name	Genomic Position (bp)	Gene Length (bp)	CDS Length (bp)	Exon	Protein Length (Amino Acids)	Molecular Weight (kDa)	Isoelectric Point (pI)	Sub-Cellular Localization
BnaA01T0032600ZS	*BnCAT1*	A01:1801796-1803991 +	2195	1488	4	495	57.30	6.5	Cytoskeleton
BnaA03T0547900ZS	*BnCAT2*	A03:31026573-31028922 −	2349	1479	5	492	56.84	6.63	Peroxisome
BnaA06T0144600ZS	*BnCAT3*	A06:8685726-8691515 +	5789	2925	12	974	112.30	6.78	Peroxisome
BnaA07T0132000ZS	*BnCAT4*	A07:17121532-17124087 −	2555	1479	5	492	56.90	6.83	Cytoplasm
BnaA07T0132100ZS	*BnCAT5*	A07:17124704-17130749 −	6045	1671	6	556	64.83	8.42	Chloroplast
BnaA08T0131200ZS	*BnCAT6*	A08:18424311-18426301 +	1990	1470	4	489	565.40	6.9	Peroxisome
BnaA08T0247000ZS	*BnCAT7*	A08:25020319-25023539 −	3220	1479	5	492	56.80	7.69	Peroxisome
BnaC03T0740800ZS	*BnCAT8*	C03:71851456-71853466 +	2010	1470	4	489	56.52	6.77	Peroxisome
BnaC05T0176000ZS	*BnCAT9*	C05:11786858-11795441 +	8583	2949	12	982	113.09	6.94	Mitochondrion
BnaC07T0194100ZS	*BnCAT10*	C07:32771906-32781676 −	9770	3123	15	1040	120.71	7.34	Chloroplast
BnaC07T0524400ZS	*BnCAT11*	C07:59429784-59432167 −	2383	1479	4	492	56.78	6.63	Peroxisome
BnaC08T0260800ZS	*BnCAT12*	C08:34921906-34925039 +	3133	1479	5	492	56.80	7.69	Peroxisome
Bnascaffold0025T0038400ZS	*BnCAT13*	0025:3787780-3790910 −	3130	1434	5	477	55.19	7.72	Peroxisome
Bnascaffold0026T0027200ZS	*BnCAT14*	0026:2854478-2857610 +	3132	1479	5	492	56.80	7.69	Peroxisome

In the genomic position, the positive (+) and negative (−) signs indicate the existence of a gene on the positive and negative strand of that specific marker, respectively.

## Data Availability

The datasets used and/or analyzed during the current study are available from the corresponding author on reasonable request. However, most of the data is shown in Supplementary files.

## Supplementary Materials

The following are available online at https://www.mdpi.com/article/10.3390/ijms22084281/s1, Table S1: The information of primer used in this study for gene expression analysis in qRT-PCR, Table S2: The *CAT* family genes in *Arabidopsis thaliana*, *Brassica oleracea*, and *Brassica rapa*, Table S3: The information of gene duplication type, Ka, Ks, and Ka/Ks ratio values of *B. napus* and other three plant species, Table S4: The information of identified 8 motifs in BnCAT proteins, Table S5: Information of hormone- and stress-related *cis*-elements detected in the promoters regions of *BnCATs*, Table S6: The information of miRNA targeted *BnCAT* genes, Table S7: The GO enrichment analysis of 14 *BnCAT* genes.
