# Supplementary material for: Catalase (CAT) Gene Family in Rapeseed (Brassica napus L.): Genome-Wide Analysis, Identification, and Expression Pattern in Response to Multiple Hormones and Abiotic Stress Conditions
Source: Int J Mol Sci. 2021 Apr 20;22(8):4281. doi: 10.3390/ijms22084281 (PMC8074368; doi:10.3390/ijms22084281)
Supplement: Supplementary file 1 [file ijms-22-04281-s001.zip › Table S1.pdf]

Table S1. The information of primer used in this study for gene expression analysis in qRT-PCR.

**Gene ID**	**Gene name**	**Forward primer**	**Reverse primer**
BnaA01T0032600ZS	BnCAT1	GCTTGCCTTCTGTCCTGCTA	CGGTGTCTCTGAGTATCGGC
BnaA03T0547900ZS	BnCAT2	GCTTGCCTTCTGTCCTGCTA	GTCCAAGACGGTGTCTCTGG
BnaA06T0144600ZS	BnCAT3	TTTCGACCCGCTTGATGTGA	AGACCTGGGTTGAAAGCGAG
BnaA07T0132000ZS	BnCAT4	GGCCTGAAGATGTCTTGCCT	AGGAACCACAATAGCAGGGC
BnaA07T0132100ZS	BnCAT5	TTCAAGCAGCCAGGAGACAG	CCAGATGCTGCGGATCTCAT
BnaA08T0131200ZS	BnCAT6	GCTAAATCCCTGAATCTCTCCA	GACGAAGAGGTGATAGACAGAG
BnaA08T0247000ZS	BnCAT7	AGCTTGCTTTCAACCCTGGT	GATGTCTCTGCGTGTCACCA
BnaC03T0740800ZS	BnCAT8	CCTTATCTGACCCCCGCATC	TGGTCTCACGTTCAGATGGC
BnaC05T0176000ZS	BnCAT9	CATCTCGTCCCTCACCATCG	GATTCCTCTGGCGTGAACCA
BnaC07T0194100ZS	BnCAT10	GCCGAACCCGAAAACAAACA	GTGGACACCGAAACCTTCCA
BnaC07T0524400ZS	BnCAT11	GCTAGAGGAGCCAGTGCAAA	TCGGGACTTCCACGTTCATG
BnaC08T0260800ZS	BnCAT12	AGCTTGCTTTCAACCCTGGT	CATTCACAGGCAGCTGGAGA
Bnascaffold0025T0038400ZS	BnCAT13	TCTCCAGCTGCCTGTGAATG	TAGGAACTTTCTCGGCGCAG
Bnascaffold0026T0027200ZS	BnCAT14	TTTCGACCCGCTTGATGTGA	CACCACAAGACCAGGGTTGA
